# The stillbirth ‘scandal’

**DOI:** 10.1186/1471-2393-15-S1-A11

**Published:** 2015-04-15

**Authors:** Ruth C Fretts

**Affiliations:** 1 Harvard Vanguard Medical Associates, Boston, Massachusetts , USA

## 

The scope of stillbirth has been overlooked by many, few would estimate that in high income countries that, late stillbirths (pregnancies 28 weeks or later) occurs twice as often as death due to HIV/ AIDS; ten times more common than deaths due to Hepatitis B; twice a common as deaths due to congenital anomalies; twice as common as deaths due to preterm complications, and ten times more common that Sudden Infant Deaths (SIDS) [1].

Perinatal audit is the key to identifying potentially modifiable factors that contribute to stillbirth: higher than expected intrapartum deaths should trigger a review of labor and delivery procedures; higher than expected number of losses of multiples should trigger a review of advanced reproductive technologies services [2]. Stillbirth prevention strategies in developed countries do share some similarities to those in developing countries, for example ensuring that poor and less educated women have access to contraception, timely access to good prenatal care. The most demonstrable effect of early prenatal care is the accurate dating of the pregnancy, screening for infection and the prenatal screening for chromosomal and congenital anomalies. In a setting where there is the availability of abortion for affected pregnancies, the number of stillbirths related to anomalies can be reduced significantly [3].

Suboptimal care has been shown to occur in 10 to 60% of stillbirths in developed countries [2]. The most common errors are failure to identify emerging clinical disorders, (such as fetal growth restriction), failure to use up-dated “best practice protocols”, poor communication or non-compliance of the members of the team (including those responsible to follow up with patients when appointments are missed).

By focusing some light on the problem of stillbirth there has evolved a number of new and potentially helpful observations. The appreciation that advanced maternal age, racial minority (specifically within the United States non-Hispanic black status), and severe obesity all are associated with an increased risk of stillbirth after 39 weeks of gestation, providers have the opportunity to either increase fetal surveillance or offer induction, thus treating these women as post-dates sooner than their low risk peers. A program of increasing awareness of the importance of fetal movement as well as the optimal management of decreased fetal movement has been shown to reduce the overall stillbirth rate by 30%. [4].

Other interesting recent observations that may modify stillbirth risk are that that the habit of left-lying during sleep may reduce the risk of late pregnancy [5]; that significant maternal stress has been associated with and increased risk of stillbirth [6], and that the evolution of genetic testing to include the evaluation of microarrays (which detects a single-nucleotide polymorphism or duplications or deletions of 500kb or greater) is more sensitive than standard karyotype to a detect potential cause of stillbirth [7].

Hopefully with on-going research we will develop a greater understanding of the elephant in the room and fewer parents will end up in the “stillbirth club”.
